# Mechanism of interactions between α-conotoxin RegIIA and carbohydrates at the human α3β4 nicotinic acetylcholine receptor

**DOI:** 10.1007/s42995-021-00108-9

**Published:** 2021-07-22

**Authors:** Meiling Zheng, Han-Shen Tae, Liang Xue, Tao Jiang, Rilei Yu

**Affiliations:** 1 Molecular Synthesis Center & Key Laboratory of Marine Drugs, Ministry of Education, School of Medicine and Pharmacy, Ocean University of China, Qingdao, 266003 China; 2 Innovation Platform of Marine Drug Screening & Evaluation, Pilot National Laboratory for Marine Science and Technology (Qingdao), Qingdao, 266100 China; 3 Illawarra Health and Medical Research Institute (IHMRI), University of Wollongong, Wollongong, NSW 2522 Australia; 4 Laboratory for Marine Drugs and Bioproducts, Pilot National Laboratory for Marine Science and Technology (Qingdao), Qingdao, 266003 China

**Keywords:** Oligosaccharide chains, nAChR, Conotoxin, Action mechanism

## Abstract

Conotoxins are marine peptide toxins from marine cone snails. The α-conotoxin RegIIA can selectively act on human (h) α3β4 nicotinic acetylcholine receptor (nAChR), and is an important lead for drug development. The high-resolution cryo-electron microscopy structure of the α3β4 nAChR demonstrates several carbohydrates are located near the orthosteric binding sites, which may affect α-conotoxin binding. Oligosaccharide chains can modify the physical and chemical properties of proteins by changing the conformation, hydrophobicity, quality and size of the protein. The purpose of this study is to explore the effect of oligosaccharide chains on the binding modes and activities of RegIIA and its derivatives at hα3β4 nAChRs. Through computational simulations, we designed and synthesized RegIIA mutants at position 14 to explore the importance of residue H14 to the activity of the peptide. Molecular dynamics simulations suggest that the oligosaccharide chains affect the binding of RegIIA at the hα3β4 nAChR through direct interactions with H14 and by affecting the C-loop conformation of the binding sites. Electrophysiology studies on H14 analogues suggest that in addition to forming direct interactions with the carbohydrates, the residue might play an important role in maintaining the conformation of the peptide. Overall, this study further clarifies the structure–activity relationship of α-conotoxin RegIIA at the hα3β4 nAChR and, also provides important experimental and theoretical basis for the development of new peptide drugs.

**Supplementary Information:**

The online version contains supplementary material available at 10.1007/s42995-021-00108-9.

## Introduction

Conotoxins are marine peptide toxins isolated from the venom of cone snails which are used for prey capture and/or predator defense. Based on the conservation of their genes and their precursor protein signal peptides, they can be divided into A, M, P, O, S, T and other superfamilies (Robinson and Norton 2014). Conotoxins are processed from precursor peptides containing 70–120 amino acid residues (Olivera 2006) and, mature peptides generally contain 12–13 amino acids and multiple pairs of disulfide bonds. Among them, the α-conotoxins (α-Ctxs) are selective antagonists for various nicotinic acetylcholine receptor (nAChR) subtypes (Azam and McIntosh 2009; Olivera et al. 2008). The α-Ctxs are a family of small peptides rich in disulfide bonds, which are composed of 12–20 amino acids. The α-Ctxs are divided in two loops (m and n) with the number of residues in each loop is used to categorize them into several structural subgroups (m/n) and the loop size roughly correlates with the pharmacological target selectivity (Lebbe et al. 2014).

The nAChRs are ligand-gated ion channels that contain functional cysteine loops in their sequence and belong to the cys-loop receptor family (Taly et al. 2009). This family of receptors is derived from a common gene and, other members include the serotonin (5-HT3), γ-aminobutyric acid type A (GABA_A_) and glycine receptors (Changeux 2012). The nAChRs are pentameric transmembrane proteins composed of five subunits and, in vertebrates there are at least 17 nAChR subunits including α1–α10, β1–β4, γ, δ and ε (Gotti et al. 2007). Different subunits aggregate to form nAChR subtypes with different functions (Gotti and Clementi 2004). The receptor consists of an extracellular domain (ECD), a transmembrane domain (TMD) and an intracellular domain (ICD). The ECD of two adjacent subunits contributes to the ligand binding site, consisting of loops A, B, and C of the principal subunit and the β-sheet of the complementary subunit.

The nAChRs are divided into two categories: muscle and neuronal types. The α3β4 nAChRs are mainly expressed on the sensory and autonomic nerve central cells of the peripheral nervous system, and on the neurons of the central nervous system (Sharma 2013). Studies have suggested the α3β4 subtype does not only mediate nicotine withdrawal, but it also reduces self-administration of many drugs of abuse (Glick et al. 2008; Jackson et al. 2013, 2015). In addition, α3β4 nAChRs are overexpressed in tumor cells and play a role in promoting carcinogenesis upon binding of ligands (i.e., nicotine) (Yi et al. 2012). Therefore, the discovery of high-efficiency and low-toxicity α3β4 nAChR inhibitors could be developed as lead compounds for the treatment of these major diseases (Posadas et al. 2013; Qian et al. 2019).

RegIIA is a 4/7-α-Ctx isolated from the venom of *Conus regius*, and potently inhibits the human α3β2, α3β4 and α7 nAChRs (Franco et al. 2012) with IC_50_ of 130, 50 and 210 nmol/L, respectively (Cuny et al. 2016). RegIIA is more potent at murine α3β2 than human α3β2, but the potency of RegIIA at the α3β4 subtype of the two species is similar. Previous studies have confirmed that Glu98 of the murine α3 subunit (Pro at the human α3 subunit) is mainly responsible for the species potency gap (Kompella et al. 2015a). Through rational design of analogues, [N11A, N12A]RegIIA has 27-fold improved selectively for hα3β4 subtype than hα3β2, despite sevenfold loss of potency compared to the parent peptide (Kompella et al. 2015b).

**Fig. 1:**
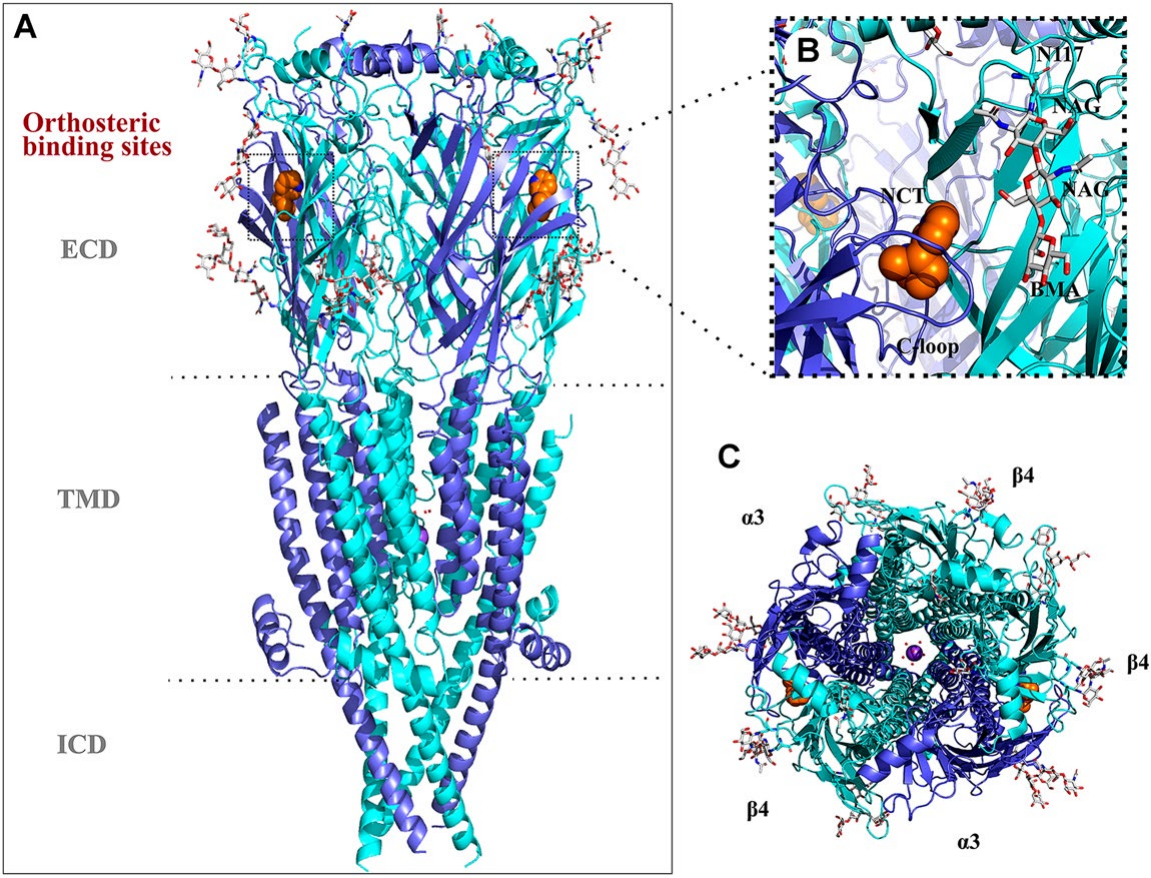
Structure of the human (h)α3β4 nAChR (PDB ID: 6PV7). **A** Side view of the hα3β4 nAChR bound with two nicotine (orange). **B** A magnified view of the orthosteric binding site (dashed frame). The silver white colored oligosaccharide chain is composed of two β-D-glucosamine (NAG) and one β-D-mannose (BMA), which are connected to the receptor asparagine residue 117 through N-glycosidic bonds. **C** Top view of the hα3β4 nAChR. The α3 and β4 subunits are colored purple and cyan, respectively

In 2019, Gharpure et al. successfully resolved the first structure of hα3β4 nAChR bound with nicotine through cryo-electron microscopy, with a resolution of 3.34 Å (Fig. 1A) (Gharpure et al. 2019). A large number of carbohydrates are observed at the apex of the ECD and near the orthosteric binding sites, and each carbohydrate is composed of two or three monosaccharides in a different order. The linear oligosaccharides near the orthosteric binding site are composed of two β-d-glucosamine and one β-d-mannose (Fig. 1B).

It is generally agreed that α-Ctxs are selective antagonists of nAChRs, and its site of action is located on the orthosteric binding site. Thus, we proposed that this linear oligosaccharide extends to the binding site of conotoxins, which may potentially affect conotoxin binding and its activity. In addition, the linear oligosaccharides are located close to the C-loop (Fig. 1B, C), thus they may also influence the receptor-conotoxin interaction by affecting the conformation of the loop. So far, influence of carbohydrates on conotoxin-nAChR dynamics is yet to be investigated. Therefore, in this study, computational simulations and two-electrode voltage clamp electrophysiology were used to explore whether carbohydrates on hα3β4 nAChRs can affect the interactions with RegIIA and its analogues. Our findings suggest the oligosaccharide chains not only facilitate the interaction of RegIIA residue H14 with the receptor, they also affect C-loop conformation. Additionally, electrophysiology results support direct interaction of residue H14 with the carbohydrates and it may also play a role in maintaining the conformation of RegIIA. This study contributes to understanding the structure–activity relationship of RegIIA at the hα3β4 nAChR, and assists in the design of novel drugs targeting the α3β4 subtype.

## Results and discussion

### Synthesis of α-conotoxin RegIIA and its analogues

**Fig. 2:**
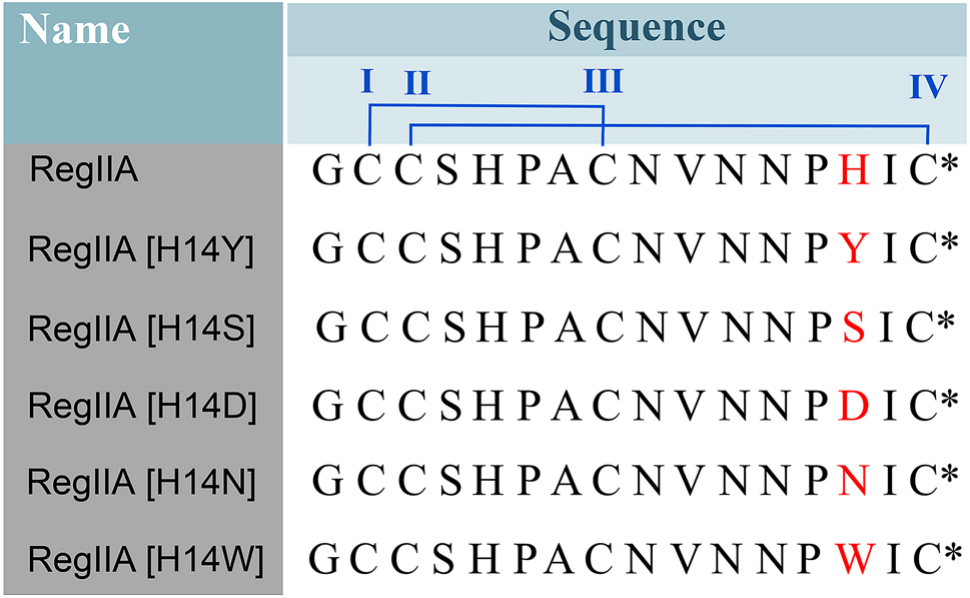
Amino acid sequence of α-Ctx RegIIA and H14 analogues. Residues at 14 position are colored red, and the disulfide bonds are colored blue. RegIIA and its derivatives contain two disulfide bonds (I–III and II–IV) (*represents amidated C-terminus)

RegIIA and its analogues (Fig. 2) were synthesized by solid-phase Fmoc chemistry, as previously described (Yu et al. 2013). To achieve regioselective oxidation, Fmoc-Cys(Trt)-OH was used at Cys I and Cys III positions, and Fmoc-Cys(Acm)-OH was used at Cys II and Cys IV positions. The fully oxidized product was separated and purified by RP-HPLC (reversed-phased-high performance liquid chromatography). The molecular weight and purity were confirmed by electrospray ionization–mass spectrometry (ESI–MS) and analytical HPLC (Supplementary Fig. S1).

### Molecular dynamics simulations of RegIIA at hα3β4 nAChRs.

**Fig. 3:**
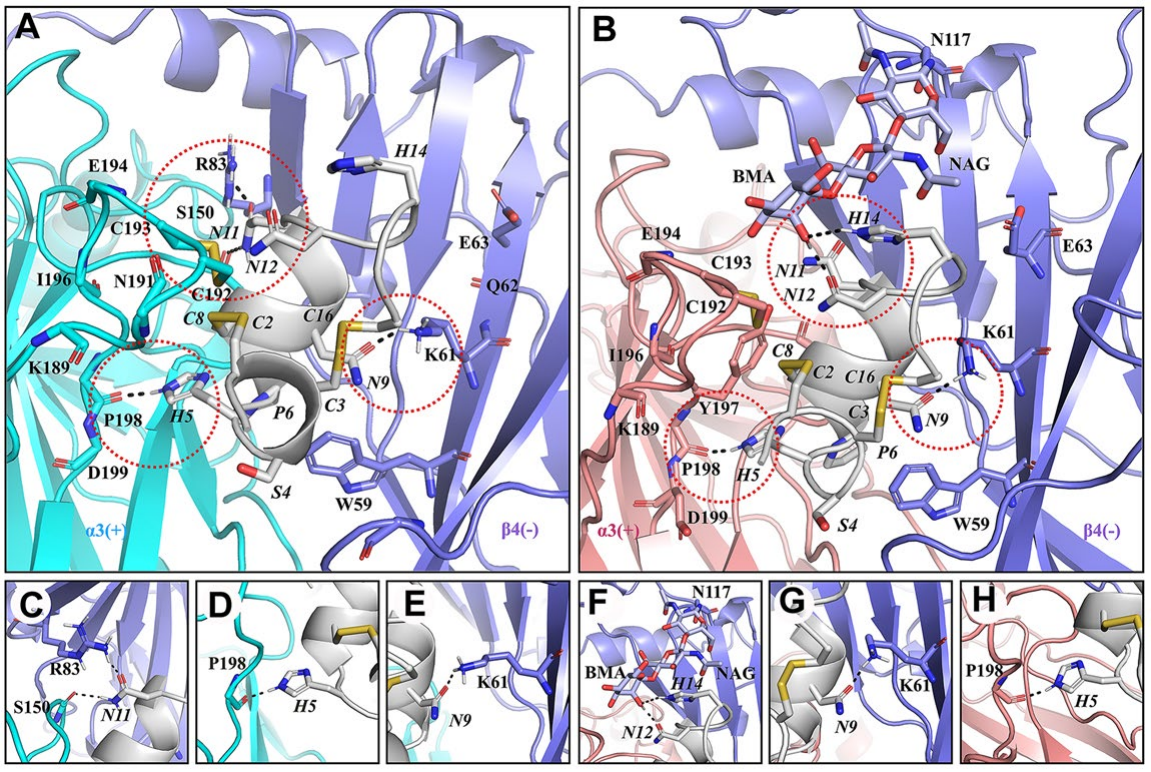
Binding modes of RegIIA at the binding site of hα3β4 nAChR. **A** In the carbohydrate-free model, several hydrogen bonds (dashed lines) are formed between pairs of interacting residues. The α3( +) interface is shown in cyan, β4(−) in purple, and RegIIA in silver. **B** In the glycoprotein model, the interaction mode is similar to the carbohydrate-free model, except that β-d-mannose forms hydrogen bonds with N12 and H14 (dashed lines). The α3( +) interface is shown in peach, β4(−) in purple, and RegIIA in silver. Residues from the receptor and RegIIA are labeled using normal and italic fonts, respectively. The key interaction sites responsible for the binding of RegIIA are highlighted with dotted circles. **C**–**H**, magnification of the key sites in carbohydrate-free model (**C**–**E**) and glycoprotein model (**F**–**H**) highlighted with circles in (**A**, **B**)

We performed molecular dynamics (MD) simulations on the α-Ctx RegIIA and hα3β4 nAChR complex in the presence or absence of oligosaccharide chains. As the carbohydrates are located at the top of the receptor, only the hα3β4 nAChR ECD model was simulated. MD simulations suggested the interaction modes of RegIIA at apo- and carbohydrate-bound hα3β4 nAChRs were similar to the binding mode of RegIIA predicted in our previous studies (Fig. 3A, B, Table 1) (Cuny et al. 2016; Xu et al. 2020). In both models, RegIIA N9 and H5 residues formed hydrogen bonds with β4 K161 and α3 P198, while N11 formed direct interactions with α3 S150 and β4 R83 (Fig. 3C–E, G, H).

**Table 1:** Pairwise interactions between the RegIIA and the hα3β4 nAChR

Residue^a^	Absence of oligosaccharide chain		Presence of oligosaccharide chain	
	+ ^b^	−^c^	+ ^b^	−^c^
S4	–	D173	K145	W59, D173
H5	Y93, S148, G147, Y190, Y197, P198	–	Y93, S148, D199, Y190, Y197, P198	W59
P6	S148, G147, W149	W59, L123	Y93, S148, W149	W59, L123
A7	S148, W149, Y197, S150, Y151	R83	Y93, S148, W149 Y197, S150, Y151	–
N9	–	K61, W59, L123	–	K61, W59, L123
V10	W149, S150	N111, I113, L121, W122, L123	W149, S150	K61, I113, L121, W122, L123
N11	S150, Y197	R83, I113, L121	S150, Y197	R83,L121, R115
N12	C193, E195, Y197	–	C193, Y197	K61, **BMA**
H14	–	–	–	**BMA**, **NAG**
I15	C192, C193	–	C192, C193	**BMA**

BMA and NAG oligosaccharides are in bold

^a^Residues of RegIIA forming direct contact with the hα3β4 nAChR are listed. Contacts between hα3β4 nAChR and RegIIA are defined as van der Waals interactions when the distance between heavy atoms of RegIIA and hα3β4 nAChR is less than 4 Å. Residues of the hα3β4 nAChR and oligosaccharides forming hydrogen bonds with RegIIA are underlined

^b^Residues from the principal subunit

^c^Residues from the complementary subunit

**Fig. 4:**
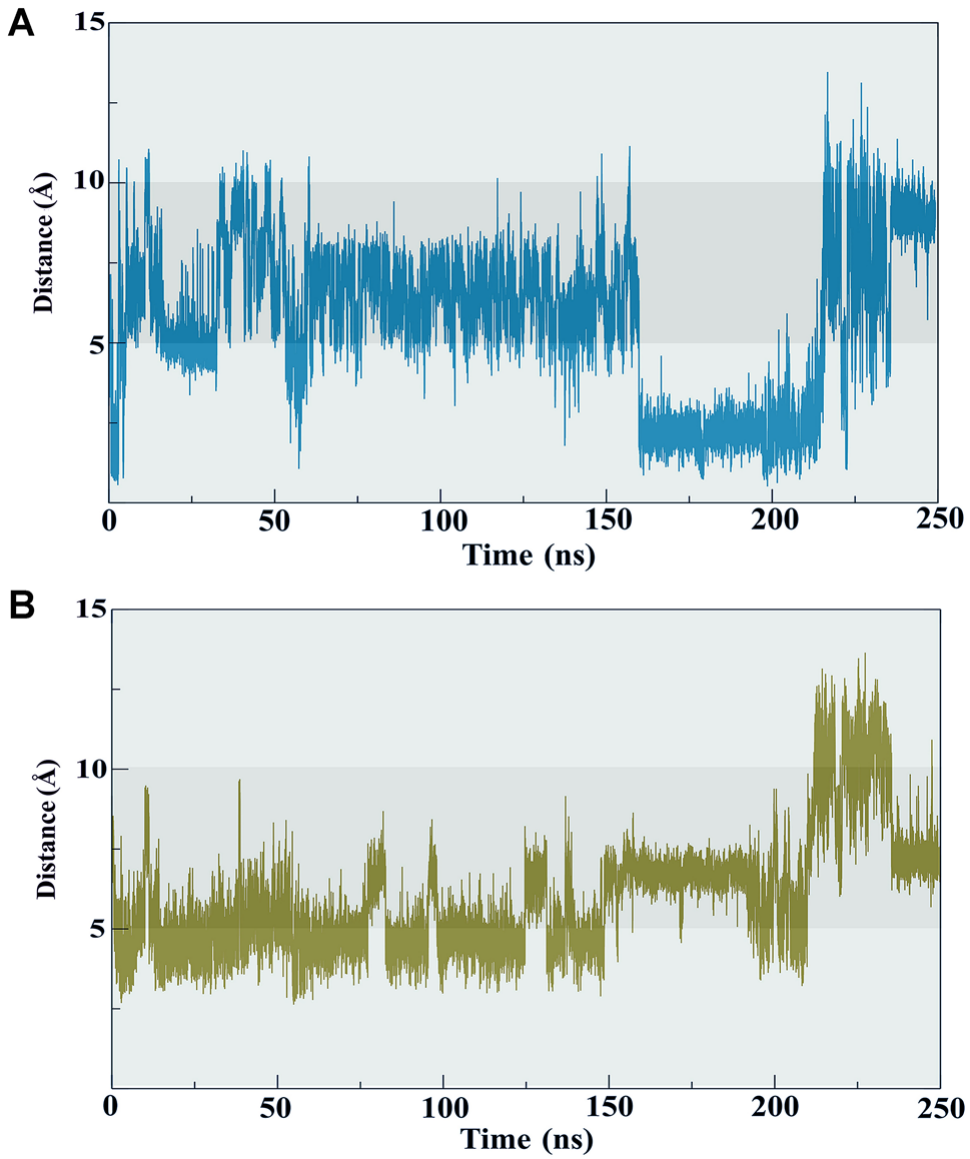
The distance between RegIIA H14 and β-d-mannose (at β4-N117 position), and β-d-glucosamine (at β4-N117 position) during MD simulations. **A** Change of the distance between the ND1 of H14 and the O2 of β-d-mannose (cyan). During 150–200 ns, the average distance between two heavy atoms is 2.5 Å, suggesting a direct contact between β-d-mannose and H14 during this period. **B** Change of distance between O3 of β-d-glucosamine and NE of H14 (orange). Before 150 ns, the interaction between β-d-glucosamine and H14 was via van der Waals force

One main difference between the two systems was the interaction between the oligosaccharide chain and β4 N117 through N-glycosidic bonds. The presence of linear oligosaccharides mainly affected RegIIA N12 and H14, which involved hydrogen bond interactions between β-d-mannose and the two residues (Fig. 3B, F). The 250 ns-MD simulation also showed change of the distance between β-d-mannose and N12 and H14, (Fig. 4A, B, Supplementary Fig. S2) and although the contact was unstable, interactions with N12 was relatively more stable than H14. Overall, MD simulations indicated that the linear oligosaccharide at β4 N117 positions was flexible and formed relatively weak interactions with residues at the solvent exposed surface of RegIIA.

**Fig. 5:**
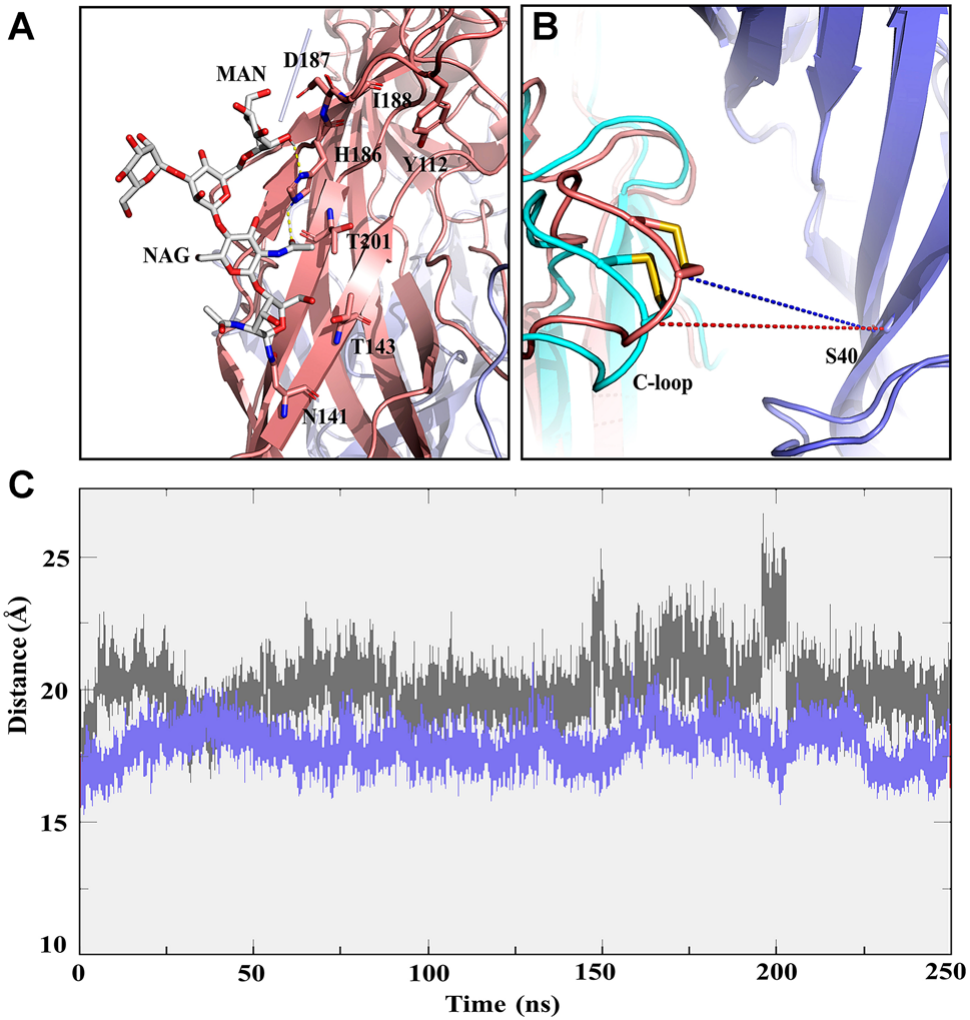
Effects of the carbohydrate at α3 N141 on the conformation of the binding site. **A** H186 of α3 forms hydrogen bonds with β-d-glucosamine and α-d-mannose on the oligosaccharide chain. The α3 interface is shown in peach, β4 in purple, and oligosaccharide chain in silver white. **B** Superimposition of carbohydrate-free model (α3 interface is shown in cyan and β4 in purple) and glycoprotein model (α3 interface is shown in peach and β4 in purple).The distance used to characterize C-loop opening in the hα3β4 nAChR is measured between the CA of α3 C193 and the CA of β4 S40. The red and blue dashed lines indicate the distance of C-loop opening in the glycoprotein model and the carbohydrate-free model, respectively. **C** The 250 ns MD simulation of carbohydrate-free (red) and -bound (black) models showing change of the distance between the CA of α3 C193 and the CA of β4 S40

We also found that the oligosaccharide chain linked to α3 N141 through N-glycosidic bonds can directly interact with α3 H186 (Fig. 5A, Supplementary Fig. S3). The average distance between the CA of α3 C193 and the CA of β4 S40 on the C-loop in apo- and carbohydrate-bound models was about 18 Å and 20 Å, respectively (Fig. 5C). Similarly, the C-loop was also slightly more opened in the carbohydrate-free model than the carbohydrate-bound model (Supplementary Fig. S7). The more closed C-loop suggested that in the presence of carbohydrates, RegIIA had more close contacts with the binding site, especially with the C-loop (Fig. 5B). Therefore, we speculated that the presence of linear oligosaccharide chains did not only affect the binding mode of RegIIA at the hα3β4 nAChR binding pocket, but also had a slight impact on C-loop conformation.

### Effect of RegIIA H14 analogues at hα3β4 nAChRs

Through MD simulations, we proposed that H14 of RegIIA played a vital role in interactions with the carbohydrate at β4 N117. Thus, to explore H14 contribution to RegIIA activity at the hα3β4 subtype, H14 RegIIA analogues were synthesized and tested at heterologous hα3β4 nAChRs expressed in *Xenopus laevis* oocytes.

**Fig. 6:**
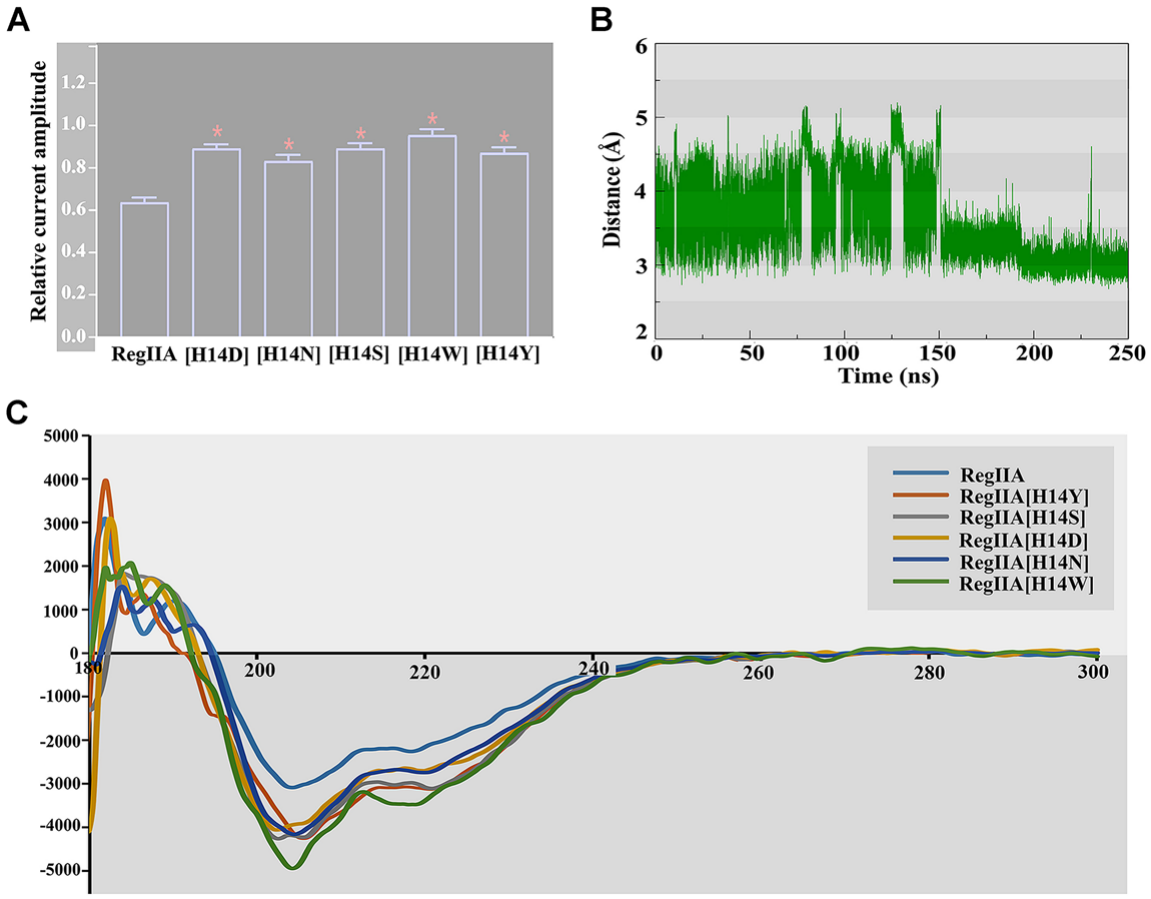
Mutational effects at the activity of RegIIA. **A** Bar graph of RegIIA and analogues (50 nmol/L) inhibition of ACh-evoked peak current amplitude mediated by hα3β4 nAChRs (*P < 0.0001 compared to RegIIA). Whole-cell currents were activated by 300 μmol/L ACh (mean ± SEM, *n* = 6–12). **B** The 250 ns MD simulation showing change of the distance between the N of H14 and the ND1 of H14 (green). The average distance between two heavy atoms is 3.5 Å. **C** Circular dichroism spectra of RegIIA and its analogues

Due to the close contact between H14 and the carbohydrate at β4 N117, residues with varied physico-chemical properties, such as hydrophilic or aromatic residues were introduced to position 14 of RegIIA. All analogues had substantially decreased potency in comparison to the wild-type RegIIA (Fig. 6A). Therefore, we speculated that the function of H14 may be unique. In addition to direct interactions with the carbohydrate, H14 might also play an essential role at sustaining the conformation of the peptide. Indeed, in MD simulations, the side chain of H14 formed an intraresidue H-bond (Fig. 6B, Supplementary Fig. S4), which contributes to maintaining the three-dimensional structure of RegIIA. Through circular dichroism analysis, we also identified that H14 is essential to the secondary structure of the peptide, and replacement of H14 resulted in certain degree of shift at 200–220 nmol/L (Fig. 6C).

In consideration of the direct interactions between the carbohydrate and H14, removal of the carbohydrate from β4 N117 might explain decrease the potency of RegIIA. Indeed, in our previous study RegIIA was sixfold less potent at the β4 N117D mutant (Cuny et al. 2016). However, the significant activity decrease was not likely resulted from β4 N117 direct interactions due to the long distance between β4 N117 and RegIIA H14. Thus, it is reasonable to deduce that the decrease is more likely due to the removal of the carbohydrate rather than from the change of the side chain interactions with the toxin.

## Conclusions

This study explored the effect of oligosaccharide chains on the binding mode of RegIIA at hα3β4 nAChRs, and the mutational effects at position 14 of RegIIA on the inhibitory activity of the peptide at hα3β4 nAChRs. The oligosaccharide chains interacted with N12 and N14 of RegIIA through unstable hydrogen bond interactions due to the flexibility of the sugar chain itself. Subsequent research suggested that the oligosaccharide chains also affected C-loop opening by inducing the C-loop to move inwards, thus reducing the opening. From electrophysiology experiments on heterologous hα3β4 nAChRs, we proposed the functional uniqueness of H14 and its importance for the inhibitory activity of the peptide at hα3β4 nAChRs. Overall, this study further clarifies the structure–activity relationship of α-Ctx RegIIA, and provides important experimental and theoretical basis for the development of new peptide drugs.

## Materials and methods

### Homology modeling

An initial model of the complex of RegIIA and hα3β4 nAChRs was established using Modeller (Sali and Blundell 1993; Webb and Sali 2017) (version 9.14), as described previously (Yu et al. 2011). The crystal structure of acetylcholine binding protein (AChBP) bound with an α-Ctx PnIA mutant (PDB identifier 2BR8 (Celie et al. 2005)) and the crystal structure of hα3β4 nAChRs (PDB identifier 6PV7) were used as templates to construct α-Ctx RegIIA and hα3β4 nAChRs complex model. The α3β4 nAChR model was composed of two α3 and three β4 subunits. Modeller was used to generate 100 model structures, and those with the lowest DOPE score according to the energy score, were used for subsequent MD simulations to further optimize the structure. For the carbohydrate-apo model, the entire hα3β4 nAChR was simulated and for the carbohydrate-bound model only the ECD was simulated. Then, Na^+^ was added to the whole system to make it appear electrically neutral and energy optimization of the whole system was carried out. First, constrained optimization was performed in solute constraining force of 100 kcal mol-1-Å, followed by 2000-step steepest descent method optimization and 3000-step conjugate gradient method optimization. After the first round of optimization, the position restriction was removed. The MD simulations process consisted of heating and equilibrium. The entire system was heated for 100 ps under equal volume conditions to gradually heat the temperature from 50 to 300 K, and the solute binding force was 5 kcal mol-1.Å-2. Subsequently, the MD simulation was performed for 250 ns under the boundary conditions of temperature and pressure maintained at 300 K and 1 atmosphere respectively (Supplementary Fig. S5).

In all dynamic simulations, Lipid14 force field (Dickson et al. 2014) was used for lipids, and ff14SB force field (Maier et al. 2015) was used for proteins and peptides. The SHAKE algorithm (Ryckaert et al. 1977) was used for hydrogen bonds where, the experimental step length in the heating process and the equilibrium process was set to 2 fs, and the PME (Darden et al. 1993) method was used to handle long-distance electrostatic interactions. The software VMD (Humphrey et al. 1996) (http://www.ks.uiuc.edu/) was used to analyze the motion trajectory after MD simulations and calculate the RMSD value of the resulting conformation.

### Synthesis of α-conotoxin RegIIA and its analogues

The chemical synthesis of peptides was as previously reported (Yu et al. 2013). Rink amide resin (loading amount 0.631 mmol/g), was saturated with a mixed solution of dimethylformamide (DMF): dichloromethane (DCM) (1:1) for 4 h, then 20% piperidine solution was used to remove the Fmoc protecting group on the resin (30 min). Coupling was carried out with five times equivalent of amino acid and 4.5 times equivalent of HCTU dissolved in appropriate amount of DMF, then 5 times equivalent of diisopropylethylamine (DIPEA) were added. Reaction was carried out at a constant temperature in a shaker for 1 h, and washed three times with DCM and DMF. The Fmoc protective group was removed with 20% piperidine solution (30 min), washed three times with DCM and DMF followed with addition of 10 ml trifluoroacetic acid: triisopropylsilane: water (9:0.5:0.5) and reacted at a constant temperature on a shaker for 3 h. The resin was washed with DCM three times, and the filtrate was combined. After removal of excess trifluoroacetic acid, four times the volume of ice ether was added to precipitate the peptide, the suspension was centrifuged for 10 min, and the supernatant was discarded to obtain a white paste-like precipitate as a crude peptide. An appropriate amount of acetonitrile:water (1:1) was used to dissolve the crude peptide, and ammonium bicarbonate was added to pH = 8. The solution was oxidized for 48 h. The oxidized product was purified by RP (reversed-phase) HPLC using a gradient of buffer A (90% water, 10% acetonitrile, 0.05% trifluoroacetic acid) and B (90% acetonitrile, 10% water, 0.05% trifluoroacetic acid) of 100% to 40% for 1 h with a flow rate 6 ml/min. Peptide was oxidized by adding 5 mg/ml iodoacetonitrile, under closed environment at 28 °C for about 2 h. Subsequently, 5 mg/ml ascorbic acid aqueous solution was added to neutralize excess iodine. The completely oxidized product was separated and purified by RP-HPLC, and the separation conditions were same as above. After the solution was lyophilized, a white powdery solid was obtained as the final product, and the product was frozen and stored at − 20 °C.

### Circular dichroism

Circular dichroism (CD) was used to qualitatively judge the secondary structure of the peptide. The peptide was dissolved in an aqueous solution of acetonitrile (acetonitrile: water = 1:1). CD was performed on a Jasco J-810 spectrophotometer at room temperature over the wavelength range of 250–190 nm using an optical path of 1.0 mm, a bandwidth of 1.0 nm, and a response time of 2 s. Then the values were averaged from three scans. Finally, the formula $\left[ \theta \right] = 1000 \cdot m\deg /\left( {l \cdot c} \right)$ was used to calculate the molar ellipticity [*θ*] of the peptide, where *m*deg is the raw CD data, *c* is the peptide molar concentration (mmol/L), and *l* is the cell path length (mm).

### *Xenopus laevis* oocyte preparation and microinjection

Oocytes (Stage V–VI Dumont’s classification; 1200–1300 μm in diameter) were removed from *X. laevis* by surgical laparotomy and defolliculated with 1.5 mg/ml collagenase Type II (Worthington Biochemical Corp., Lakewood, NJ, USA). Defolliculation was done at room temperature (21 –24 °C) for 1–2 h in OR-2 solution containing (in mmol/L) 82.5 NaCl, 2 KCl, 1 MgCl_2_, 5 HEPES, pH 7.4.

Plasmid pT7TS constructs of hα3 and hβ4 were linearized with XbaI (NEB, Ipswich, MA, USA) for in vitro T7 mMessage mMachine®-cRNA transcription (AMBION, Foster City, CA, USA). Oocytes were injected with 5 ng of hα3β4 cRNAs at α3 to β4 ratio of 1:1 (concentration confirmed spectrophotometrically and by gel electrophoresis) using glass pipettes as described previously (Arias et al. 2020).

### Two-electrode voltage clamp recording of oocytes and data analysis

Two-electrode voltage clamp recordings of *X. laevis* oocytes expressing hα3β4 nAChRs were performed 2–7 days post-cRNA microinjection at room temperature using a GeneClamp 500B amplifier and pClamp9 software interface (Molecular Devices, Sunnyvale, CA, USA) at a holding potential − 80 mV. Both voltage-recording and current-injecting electrodes were pulled from GC150T-7.5 borosilicate glass (Harvard Apparatus, Holliston, MA, USA) and gave resistances of 0.3–1 MΩ when filled with 3 mol/L KCl.

Oocytes were perfused with ND96 solution (2 ml/min), followed by three ACh applications at 300 µmol/L (half-maximal effective concentration for hα3β4 nAChR, Cuny et al. 2016) and 3-min washouts between applications. Perfusion was stopped, and oocytes were incubated with peptide for 5 min, followed by ACh plus peptide co-application with flowing ND96 solution. All peptides were tested at 50 nmol/L, close to the reported IC_50_ of wild-type RegIIA at hα3β4 nAChRs (Cuny et al. 2016). Peptide solutions were prepared in ND96 + 0.1% FBS. Oocytes were incubated with 0.1% FBS to ensure that the FBS and the pressure of the perfusion system had no effect on the nAChRs.

Pre and post peptide incubation peak current amplitudes were measured using Clampfit 10.7 software (Molecular Devices, Sunnyvale, CA, USA) and the relative current amplitude (I_ACh+peptide_/I_ACh_) was used to assess the activity of peptide at hα3β4 nAChRs. Results were analyzed by unpaired Student’s t tests (GraphPad Prism 9 Software, La Jolla, CA, USA) and values of *P* ≤ 0.05 were considered statistically significant.

## Supplementary Information

Below is the link to the electronic supplementary material.

Supplementary file1 (DOCX 551 kb)
