# Supplementary material for: Mechanism of interactions between α-conotoxin RegIIA and carbohydrates at the human α3β4 nicotinic acetylcholine receptor
Source: Mar Life Sci Technol. 2021 Jul 22;4(1):98–105. doi: 10.1007/s42995-021-00108-9 (PMC10077175; doi:10.1007/s42995-021-00108-9)
Supplement: Supplementary file 1 — Supplementary file1 (DOCX 551 kb) [file 42995_2021_108_MOESM1_ESM.docx]

**Mechanism of interactions between carbohydrates and α-conotoxin RegIIA at the human α3β4 nicotinic acetylcholine receptor**

**Supporting information**

Meiling Zheng^†a,b,d^, Han-Shen Tae^†c^, Liang Xue^a,b^, Tao Jiang^a,b^, Rilei Yu^*,a,b,d^

^a^ Molecular Synthesis Center & Key Laboratory of Marine Drugs, Ministry of Education; School of Medicine and Pharmacy, Ocean University of China, Qingdao 266003, China

^b^ Laboratory for Marine Drugs and Bioproducts, Qingdao National Laboratory for Marine Science and Technology, Qingdao 266003, China

^c^ Illawarra Health and Medical Research Institute (IHMRI), University of Wollongong, Wollongong, New South Wales 2522, Australia

^d^ Innovation Platform of Marine Drug Screening & Evaluation, Qingdao National Laboratory for Marine Science and Technology, Qingdao 266100, China.

^*^ Corresponding author: [ryu@ouc.edu.cn](mailto:ryu@ouc.edu.cn) (R.Yu)

^†^ These authors contributed equally to this work.


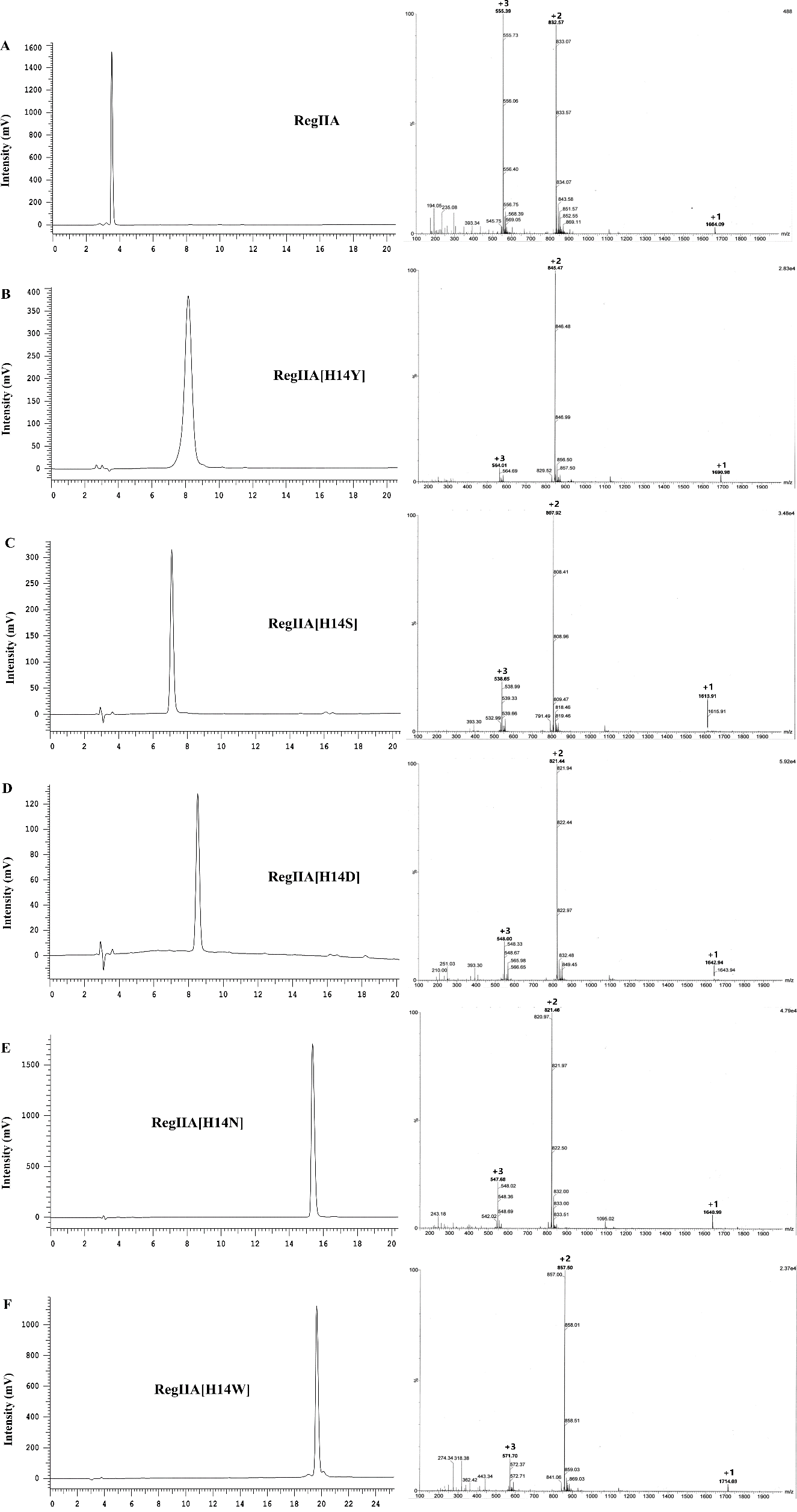


**Supplementary Fig. S1** Analytical RP-HPLC profiles and ESI-MS spectra of RegⅡA (A) and its H14 analogues (B, C, D, E and F).


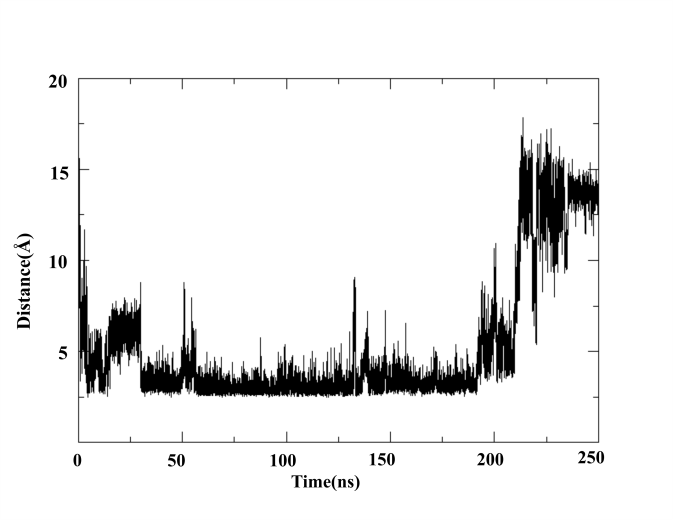


**Supplementary Fig. S2** The distance between β-D-mannose (at β4 N117 position) and RegIIA N12 during MD simulations. The average distance between two heavy atoms (OD1 of N12 and O2 of β-D-mannose) is 2.6 Å. At 50-200 ns, the H-bond between β-D-mannose and N12 is more stable than the H-bond between β-D-mannose and H14.


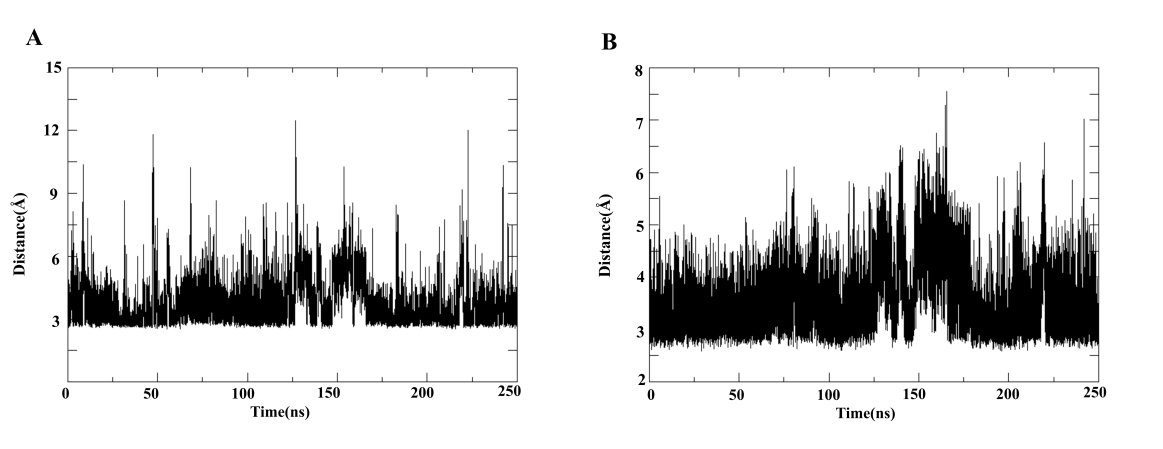


**Supplementary Fig. S3** The distance between the oligosaccharide chain at α3 N141and α3 H186, during MD simulations. (A) The average distance between the ND1 of α3 H186 and the O2 of α-D-mannose is 3.6 Å, indicating that α-D-mannose has a relatively stable contact with α3 H186 within 250 ns. (B) The 250 ns MD simulations showed change of the distance between the NE2 of α3 H186 and the O2N of α-D-mannose. The average distance between two heavy atoms is 3.5 Å, suggesting a direct contact between α-D-mannose and α3 H186 during this period


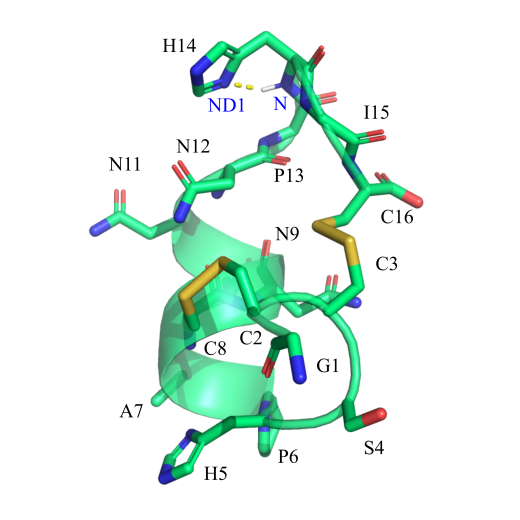


**Supplementary Fig. S4** The side chain of RegIIA H14 forms an intraresidue H-bond. Atoms from H14 are labeled in blue. The internal H-bond is represented by a yellow dashed line.


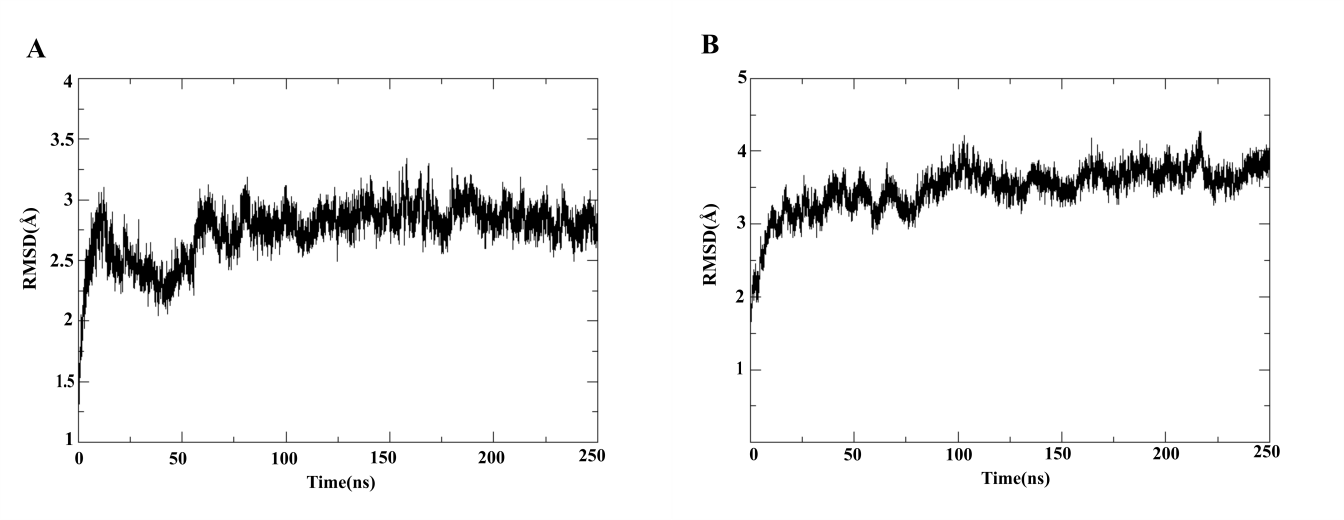


**Supplementary Fig. S5** RMSD plots of MD simulations at the hα3β4 subtype (A) carbohydrate-free and (B) glycoprotein models. The RMSD values of the two models both tend to stabilize after 50 ns at 2.5 Å and 3.5 Å for the carbohydrate-free model and the glycoprotein model, respectively.


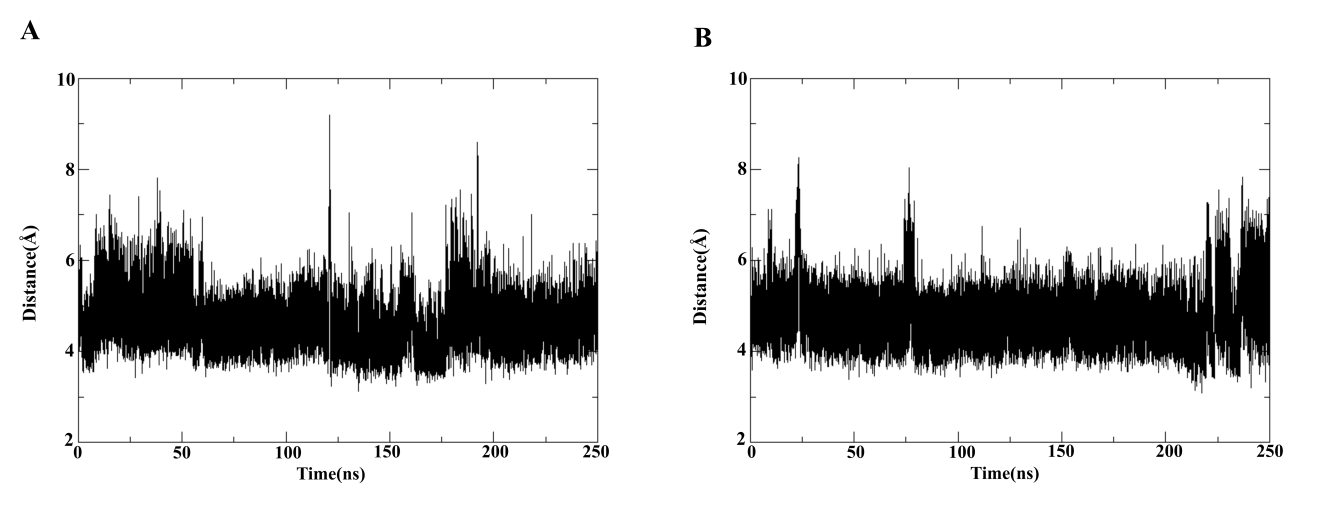


**Supplementary Fig. S6** The distance change between RegIIA P6 and β4-W59 during MD simulations. The distance between two heavy atoms (CG of RegIIA P6 and CZ2 of β4-W59) in (A) carbohydrate-free model and (B) glycoprotein model.


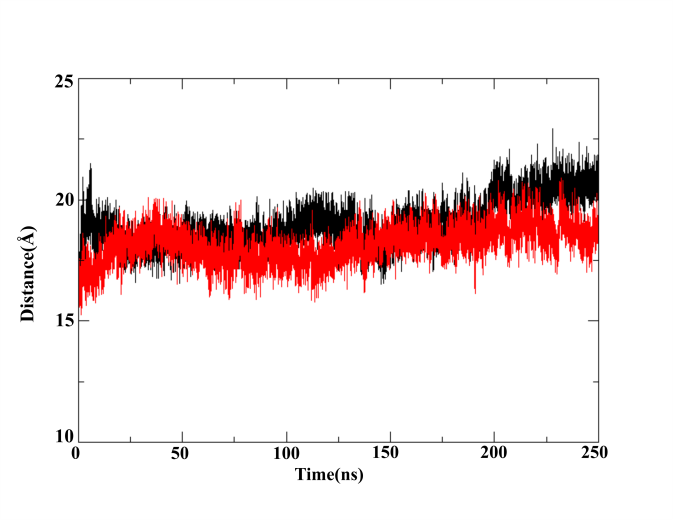


**Supplementary Fig. S7** The MD simulation of carbohydrate-free (black) and glycoprotein (red) models showed the change of distance between the CA of α3 C193 and the CA of β4 S40 in the other pocket. The C-loop is slightly more opened in the carbohydrate-free model than that of the glycoprotein model.
